# Conversion TORS after neoadjuvant immunotherapy for advanced BOT-SCC: a retrospective study

**DOI:** 10.3389/fonc.2025.1709974

**Published:** 2026-01-06

**Authors:** Quanquan Lin, Yingjuan Zhang, Jinlong Sun, Xiuli Hui, Yipeng Ren, Zhigang Song, Zhiyong Wu, Boning Cai, Lin Feng, Haizhong Zhang, Feng Wang, Qing Xi

**Affiliations:** 1 Department of Stomatology, The First Medical Center of People’s Liberation Army (PLA) General Hospital, Beijing, China; 2 Department of Stomatology, The Sixth Medical Center of People’s Liberation Army (PLA) General Hospital, Beijing, China; 3 Department of Anesthesiology, The First Medical Center of People’s Liberation Army (PLA) General Hospital, Beijing, China; 4 Department of Pathology, The First Medical Center of People’s Liberation Army (PLA) General Hospital, Beijing, China; 5 Department of Ambulatory Medicine, The First Medical Center of People’s Liberation Army (PLA) General Hospital, Beijing, China; 6 Department of Radiation Oncology, The First Medical Center of People’s Liberation Army (PLA) General Hospital, Beijing, China

**Keywords:** base-of-tongue squamous cell carcinoma, downstaging, functional outcomes, neoadjuvant immunotherapy, pembrolizumab, transoral robotic surgery

## Abstract

**Introduction:**

Advanced base-of-tongue squamous cell carcinoma (BOT-SCC) has conventionally been regarded as unsuitable for transoral resection owing to its propensity for deep invasion and the difficulty in obtaining adequate surgical margins. We evaluated whether neoadjuvant therapy could enable transoral robotic surgery (TORS) in a subset of patients with advanced BOT-SCC.

**Methods:**

In this retrospective analysis, nine consecutive patients with stage ≥T4N2bM0 BOT-SCC received three cycles of neoadjuvant therapy based on pembrolizumab, followed by TORS with concurrent neck dissection. Radiologic response and tumor shrinkage were assessed after neoadjuvant therapy. Perioperative outcomes, correlations between radiologic shrinkage and operative metrics, postoperative complications (Clavien -Dindo), and 3-month functional outcomes (MD Anderson Dysphagia Inventory [MDADI], Functional Oral Intake Scale [FOIS], and Grade, Roughness, Breathiness, Asthenia, Strain [GRBAS]) were recorded. Follow-up was conducted for oncologic outcomes.

**Results:**

Post-neoadjuvant radiologic evaluation demonstrated clinically meaningful downstaging, with partial response in 6/9 patients (66.7%) and stable disease in 3/9 patients (33.3%). Mean tumor reduction was 35.6% ± 11.2% (median, 36%; interquartile range [IQR], 28 -44). All patients achieved R0 resection. Mean operative time was 193.3 ± 46.9 min (median, 180; IQR, 165 -240), and mean intraoperative blood loss was 78.3 ± 25.4 mL (median, 70; IQR, 60 -100). Tumor shrinkage was inversely correlated with operative time (r = −0.962; 95% CI: −0.992 to −0.825; p < 0.001) and blood loss (r = −0.851; 95% CI: −0.968 to −0.430; p = 0.004), while operative time was positively correlated with blood loss (r = 0.864; 95% CI: 0.469 to 0.971; p = 0.003). No Clavien -Dindo grade ≥III complications or postoperative hemorrhage occurred. At 3 months, functional outcomes were favorable (mean MDADI 75.6 ± 10.2; FOIS ≥6 in 55.6%; median GRBAS 1.1). Over a median follow-up of 12 months, no local recurrences or distant metastases were documented.

**Discussion:**

Neoadjuvant therapy may render a subset of patients with advanced BOT-SCC eligible for TORS, enabling oncologically radical resection with low perioperative morbidity and promising early functional recovery. Prospective studies are warranted to validate patient selection criteria and to develop biomarker-guided de-intensification strategies.

## 1 Introduction

Base-of-tongue squamous cell carcinoma (BOT-SCC) is a common malignancy of the head and neck. Because of its anatomical location, conventional midline mandibulotomy approaches are highly invasive and are associated with significant postoperative morbidity, including severe impairment of swallowing and speech, which substantially reduces quality of life.

With advances in robotic surgery, transoral robotic surgery (TORS) has been increasingly adopted for oropharyngeal cancers. TORS surgery can preserve tissue due to its minimally invasive nature, which can avoid the complications associated with open procedures. However, advanced tumors remain challenging. They often show wide local extension, large volume, and deep invasion. It often requires resection of more than half of the oropharynx exposure, and achievement of safe margins is challenging, leading to increasing risk of intraoperative bleeding. Consequently, TORS has traditionally been considered contraindicated in these cases (1).

Neoadjuvant therapy is given before surgery. It may include chemotherapy, radiotherapy, targeted therapy, immunotherapy, or a combination of these. The aim is to reduce tumor burden and downstage disease to improve local control (2). Historically, induction chemotherapy was commonly used to downstage advanced head and neck squamous cell carcinoma (HNSCC), achieving tumor reduction in 60%–90% of patients and radiographic complete response (CR) in 20%–50% (3). However, evidence indicates that induction chemotherapy does not improve survival or allow for reduced surgical margins, thus limiting functional preservation (4).

The advent of immune checkpoint inhibitors has created new opportunities for neoadjuvant strategies in HNSCC. PD-1 inhibitors have shown promising efficacy in the neoadjuvant setting (5). Both monotherapy and chemo-immunotherapy produce substantial tumor shrinkage and meaningful pathologic responses in locally advanced disease (5, 6). The phase III KEYNOTE-689 trial showed that perioperative pembrolizumab improved event-free survival and increased pathologic response rates (7). Other PD-1–chemotherapy regimens have reported objective response rates (ORRs) near 90%, with pathologic complete response (pCR) in more than half of patients (8). These results suggest that immunotherapy-based neoadjuvant approaches may more effectively eradicate tumor cells and potentially eliminate occult micrometastases. It may enable meaningful downstaging and improve long-term outcomes (9).

In this study, conversion TORS denotes cases initially not amenable to oncologically safe transoral resection but rendered suitable after neoadjuvant therapy, allowing clear margins without mandibulotomy or open pharyngotomy. We retrospectively analyzed nine patients with advanced BOT-SCC and multiple nodal metastases (≥T4N2bM0). The goal was to assess whether neoadjuvant immunotherapy could downstage disease previously deemed ineligible for TORS. We evaluated conversion to minimally invasive robotic radical resection and oncologic outcomes.

## 2 Materials and methods

### 2.1 Study design

This was a single-center, retrospective, consecutive case series conducted at the PLA General Hospital between January 2022 and August 2024. The primary objective was to evaluate the feasibility and early outcomes of conversion TORS after neoadjuvant immunochemotherapy in patients with advanced BOT-SCC.

During the study period, all patients with advanced BOT-SCC discussed at the multidisciplinary tumor board (MDT) were prospectively entered into a screening log before initiation of oncologic treatment, irrespective of the eventual treatment modality or response to neoadjuvant therapy. Advanced BOT-SCC was defined at initial assessment as a T4 primary tumor with multiple nodal metastases (≥N2b).

The present analysis is restricted to consecutively screened patients who completed three cycles of neoadjuvant immunochemotherapy, met all pre-defined eligibility criteria for the neoadjuvant conversion TORS pathway, and ultimately underwent conversion TORS with curative intent together with concurrent neck dissection.

Baseline clinical and pathological stages were assigned according to the 8th edition of the AJCC/UICC TNM system. P16-positive oropharyngeal squamous cell carcinomas were staged using the HPV-mediated (p16-positive) oropharyngeal carcinoma chapter, whereas p16-negative tumors were staged using the conventional oropharyngeal carcinoma chapter (10).

#### 2.1.1 Inclusion criteria

Eligible patients were adults aged 18–80 years with histologic or cytologic confirmation of BOT-SCC before any oncologic treatment, and a clinical stage of T4 primary tumor with nodal stage ≥N2b.

#### 2.1.2 Exclusion criteria

Patients were excluded if they had received prior anticancer therapy for the index tumor (including surgery, radiation, chemotherapy, immunotherapy, or targeted therapy) or had recurrent disease after such treatments; had known or suspected distant metastasis (e.g., lung, bone, and liver); had significant comorbidities precluding neoadjuvant therapy or surgery (such as severe cardiovascular or cerebrovascular disease, hepatic or renal insufficiency, or other serious systemic illnesses); were deemed at high risk of airway obstruction that would prevent standard preoperative assessment or postoperative functional rehabilitation; or were pregnant or lactating.

#### 2.1.3 Primary endpoint

The primary endpoint was the feasibility of conversion TORS and the R0 resection rate.

#### 2.1.4 Secondary endpoints

Secondary endpoints included perioperative metrics (operative time and blood loss), complication rates (graded by Clavien–Dindo classification), functional outcomes [assessed by MD Anderson Dysphagia Inventory (MDADI), Functional Oral Intake Scale (FOIS), and Grade, Roughness, Breathiness, Asthenia, Strain (GRBAS) scales at 3 months], treatment response [radiologic per RECIST 1.1 and pathologic, including major pathologic response (MPR) and pCR], and early oncologic outcomes [disease-free survival (DFS) and overall survival (OS)].

### 2.2 Neoadjuvant regimen and timing

All patients received three 21-day cycles of neoadjuvant therapy. The regimen consisted of pembrolizumab, nab-paclitaxel, and carboplatin. Pembrolizumab (200 mg) was administered intravenously on day 1 of each cycle on a 3-weekly (Q3W) schedule. When feasible, pembrolizumab was co-administered with chemotherapy on day 1. Albumin-bound paclitaxel (nab-paclitaxel) was given at 100 mg/m² intravenously on days 1 and 8, and carboplatin (AUC 5 IV) was administered intravenously on day 1 (**Table 1**). Surgery was scheduled for a median of 14 days (IQR 12–18) after the final neoadjuvant dose, contingent upon hematologic and biochemical recovery.

**Table 1:** Neoadjuvant treatment regimen.

Component	Drug	Dose	Schedule (21-day cycle)
Immunotherapy	Pembrolizumab	200 mg	IV on day 1, Q3W
Chemotherapy	Nab-paclitaxel	100 mg/m²	IV on days 1 and 8
	Carboplatin	AUC 5	IV on day 1

Adverse events were prospectively recorded and graded according to CTCAE v5.0. Before each cycle, routine laboratories tests, including complete blood count and serum chemistry, and symptom assessments were performed. Supportive care and dose modifications were implemented as clinically indicated.

### 2.3 Imaging and response assessment

Baseline and preoperative contrast-enhanced computed tomography (CT) or magnetic resonance imaging (MRI) scans were evaluated according to RECIST 1.1. The longest diameter of solid tumors was measured, and the sum of the longest diameters (SLD) was recorded at baseline (SLD*_pre_*) and before surgery (SLD*_pos_*_t_). The percentage change was calculated as:


$$\%\Delta \text{SLD}=\frac{{\text{SLD}}_{\;post}-{\text{SLD }}_{pre}}{{\text{SLD }}_{pre}}\times 100\%$$

Whenever possible, the same imaging modality and anatomical plane were used for consistency. Two head and neck radiologists independently reviewed the scans, with any discrepancies resolved through consensus.

Although the imaging response did not alter the oncologic goal of achieving R0 resection, it informed the planning of surgical exposure, the extent of mucosal margins, and the anticipated operative time and blood loss. For cases with limited radiographic response or anticipated poor exposure, an open backup approach was prepared during the preoperative multidisciplinary conference. Concurrent neck dissection was planned based on nodal status and anatomical considerations, and its extent was not reduced solely based on RECIST response.

### 2.4 Pathologic assessment

Intraoperative frozen-section analysis was performed to evaluate surgical margins. The resected primary tumor, cervical lymph nodes, and residual margin tissue were fixed in neutral-buffered formalin, embedded in formalin-fixed paraffin-embedded (FFPE) blocks, and stained with hematoxylin and eosin (H&E) for definitive histopathologic evaluation.

Pathologists estimated the percentage of residual viable tumors (RVT%). MPR was defined as RVT ≤ 10%, and pCR was defined as the absence of residual invasive carcinoma. We conducted cross-tabulation of RECIST categories [partial response (PR) *vs*. stable disease (SD)] with MPR/pCR status and assessed the correlation between %ΔSLD and RVT%. These analyses were exploratory in nature; we report effect sizes with 95% confidence intervals (CIs) and interpret *p*-values as descriptive measures.

#### 2.4.1 Evaluation indicators

- Margin status (negative or positive).
- Residual tumor and treatment effect: viable tumor estimated in 5%. Immune-related regression features documented including tumor regression/necrosis, stromal fibrosis, foamy macrophages, and lymphocytic infiltrates.
- Histologic differentiation: graded as well, moderately, or poorly differentiated based on keratinization, intercellular bridges, and cytologic atypia.
- Invasive parameters: presence or absence of perineural invasion (PNI) and lymphovascular invasion (LVI).

#### 2.4.2 Immunohistochemistry

Immunohistochemistry was performed when residual foci were scant, morphologically atypical, or equivocal to confirm tumor cell identity and burden.

P16 expression was assessed by immunohistochemistry for all tumors. Strong, diffuse nuclear and cytoplasmic staining in ≥70% tumor cells was considered p16-positive and used as a surrogate for HPV-mediated oropharyngeal squamous cell carcinoma (11).

PD-L1 expression was evaluated by using immunohistochemistry. Membranous staining of any intensity in tumor cells and tumor-associated immune cells was recorded. PD-L1 status was summarized using the combined positive score (CPS), calculated as:


$$\text{CPS}=\frac{PD-L1-positive tumor cells+PD-L1-positive immune cells}{\text{Total viable tumor cells}}\times 100$$

#### 2.4.3 Quality control

All slides were independently reviewed by two senior pathologists who were blinded to clinical outcomes.

### 2.5 Surgical experience

#### 2.5.1 Patient counseling

We focused on patients with base-of-tongue lesions staged T4, with preoperative histologic confirmation of squamous cell carcinoma and PD-L1 positivity with a high CPS as determined by immunohistochemistry (12).

Both surgery-based management (open resection or TORS, with or without neoadjuvant immunochemotherapy) and primary concurrent chemoradiation were systematically presented as guideline-concordant standard-of-care options. All patients were fully informed of their disease status, and the indications and contraindications were thoroughly explained before treatment initiation.

The surgeon and oncologist counseled each patient in detail regarding expected oncologic outcomes, functional sequelae, and the need for adjuvant therapy. Patient preferences were documented in the medical record and were respected within the framework of oncologic safety. Patients who preferred nonsurgical management were treated with definitive chemoradiation, whereas those who elected primary open surgery underwent open resection with neck dissection.

#### 2.5.2 Neoadjuvant pathway and TORS procedure

Two weeks after the last cycle of neoadjuvant therapy, contrast-enhanced maxillofacial CT was performed (7). Only patients who demonstrated adequate tumor downstaging and were deemed technically suitable candidates for TORS, and who elected TORS rather than an open approach after shared decision-making, proceeded to conversion TORS with concurrent neck dissection.

**Figure 1:**
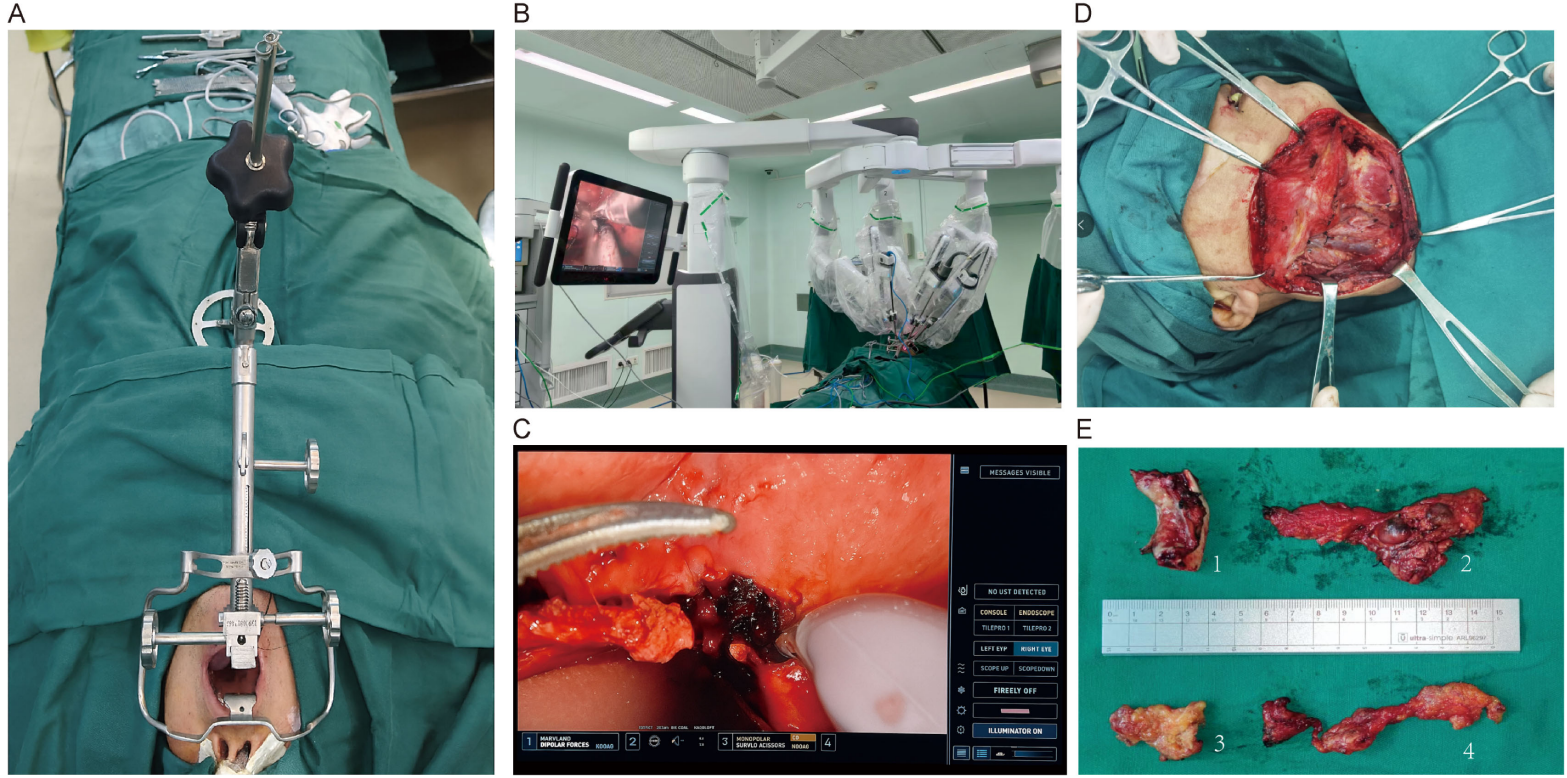
Operative workflow of TORS. **(A)** Patient positioning and transoral exposure method. **(B)** Relationship between Da Vinci Xi and patient position. **(C)** Intra-operative endoscopic field during TORS at the base of tongue showing three-sided exposure. **(D)** Cervical lymph-node dissection performed in the same session as TORS. **(E)** Specimens submitted in separate containers.

TORS was performed with the da Vinci Xi system under anesthesia. The base-of-tongue lesion was exposed via a transoral approach. After delineating the anterior tumor margin, a transverse mucosal incision was made across the tongue base, followed by a lateral pharyngeal incision along the tonsillar fossa; these were connected laterally. A midline sagittal incision on the dorsal tongue was extended to meet the lateral cuts, establishing a three-sided exposure. Dissection proceeded vertically through tongue musculature, toward the vallecula, and was tailored to the depth of invasion. Encountered lingual arteries were controlled as needed to secure hemostasis. Deep tongue muscles or mucosa involved by tumors were resected to separate the tumor from the vallecula and ensure negative margins. A concurrent cervical lymph-node dissection was performed in the same session. After resection of the primary lesion, separate margin specimens were obtained for intraoperative frozen-section analysis to confirm negative margins. The resected base-of-tongue primary and cervical lymph nodes were then submitted for routine histopathology to inform subsequent treatment planning (**Figures 1A–E**).

### 2.6 Postoperative follow-up

#### 2.6.1 Imaging and pathological examinations

Postoperative follow-up was conducted at 1, 3, and 6 months and every 3 months in 24 months. At each follow-up visit, a focused head and neck physical examination was performed. Enhanced maxillofacial CT or MRI was obtained at approximately 3 and 6 months postoperatively. Positron emission tomography (PET)-CT was performed when clinically warranted. When clinical examination or CT scan suggested a suspicious lesion, tissue confirmation was pursued by fine-needle aspiration or biopsy.

#### 2.6.2 Functional and quality-of-life assessment

##### 2.6.2.1 MD Anderson dysphagia inventory

Patient-reported swallowing-related quality of life was assessed using the MD Anderson Dysphagia Inventory (MDADI). The questionnaire was completed in clinic at 3 months after surgery. The MDADI includes one global item and three domains (functional, emotional, and physical). Each item is rated 1–5. Domain and global means are multiplied by 20 to yield a 20–100 score. Higher scores indicate better function (13).

##### 2.6.2.2 Functional oral intake scale

FOIS assesses oral intake over the prior week at 3 months. Scores range from 1 to 7. Nasogastric or gastrostomy feeding corresponds to 2–3. A soft diet corresponds to 4. Intake of two or more consistencies with special preparation or compensations corresponds to 5. Scores ≥6 indicate a clinically acceptable return to normal oral intake function (14).

##### 2.6.2.3 Grade, roughness, breathiness, asthenia, strain

Perceptual voice quality was rated at 3 months during sustained vowels and reading/spontaneous speech (≥30 s). Each parameter is scored 0–3 (0 = normal; 1 = mild; 2 = moderate; 3 = severe). Two experienced raters scored recordings independently in a quiet room. The mean of the two scores was used for analysis (15–17).

#### 2.6.3 Outcome definitions

##### 2.6.3.1 Disease-free survival

DFS is defined as the time from surgery to the first event of local, regional, or distant recurrence, or death from any cause.

##### 2.6.3.2 Overall survival

OS is defined as the time from surgery to death from any cause. Survivors were censored at last follow-up.

##### 2.6.3.3 Recurrence/metastasis adjudication

Recurrence or metastasis was adjudicated as new or progressive lesions on comparative imaging relative to pre- and post-treatment studies, with histopathologic confirmation when feasible. When biopsy was not possible, a multidisciplinary consensus integrating clinical and radiologic data was used.

### 2.7 Data analysis

Data were exported in CSV format and analyzed using R version 4.3.2 (R Foundation for Statistical Computing, Vienna, Austria). Continuous variables are summarized as mean ± standard deviation (SD) or median [interquartile range (IQR)], as appropriate; categorical variables are presented as counts and percentages. Owing to the small sample size (*n* = 9), all analyses were considered exploratory. We therefore focused on reporting effect sizes with 95% CIs and treated all *p*-values as descriptive (two-sided), without any claims adjusted for multiple testing.

For proportions, exact Clopper–Pearson CIs were calculated. For correlation analyses, both Pearson’s *r* and Spearman’s ρ are presented alongside Fisher-*z* transformed 95% CIs for transparency; the associated *p*-values are strictly descriptive. In group comparisons, the Hodges–Lehmann estimator (with CIs) was used for median differences, and Hedges’ *g* (with CIs) was applied for standardized mean differences, given the small sample size.

All graphical displays were generated using ggplot2, and statistical computations were performed using base R functions.

## 3 Results

### 3.1 Clinical characteristics

Patients for this retrospective case series spanned from January 2022 to August 2024. For the present analysis, the median follow-up from surgery among surviving patients was 12 months (range, 8–15 months).

A total of 18 patients with suspected or confirmed BOT-SCC and radiologic evidence of locally advanced disease were evaluated at the MDT. Of these, five patients did not meet eligibility criteria for the neoadjuvant conversion TORS pathway because of prior definitive treatment for the index tumor, documented distant metastases, significant comorbidities precluding neoadjuvant therapy or surgery, or a high risk of airway obstruction that would have prevented standard preoperative assessment and postoperative functional rehabilitation. Among the remaining 13 technically eligible candidates, 4 patients declined neoadjuvant therapy and/or TORS after shared decision-making and opted instead for either definitive chemoradiation or primary open surgery. These patients are reported in the screening log but are not included in the present TORS case series (**Supplementary Table 1**).

**Figure 2:**
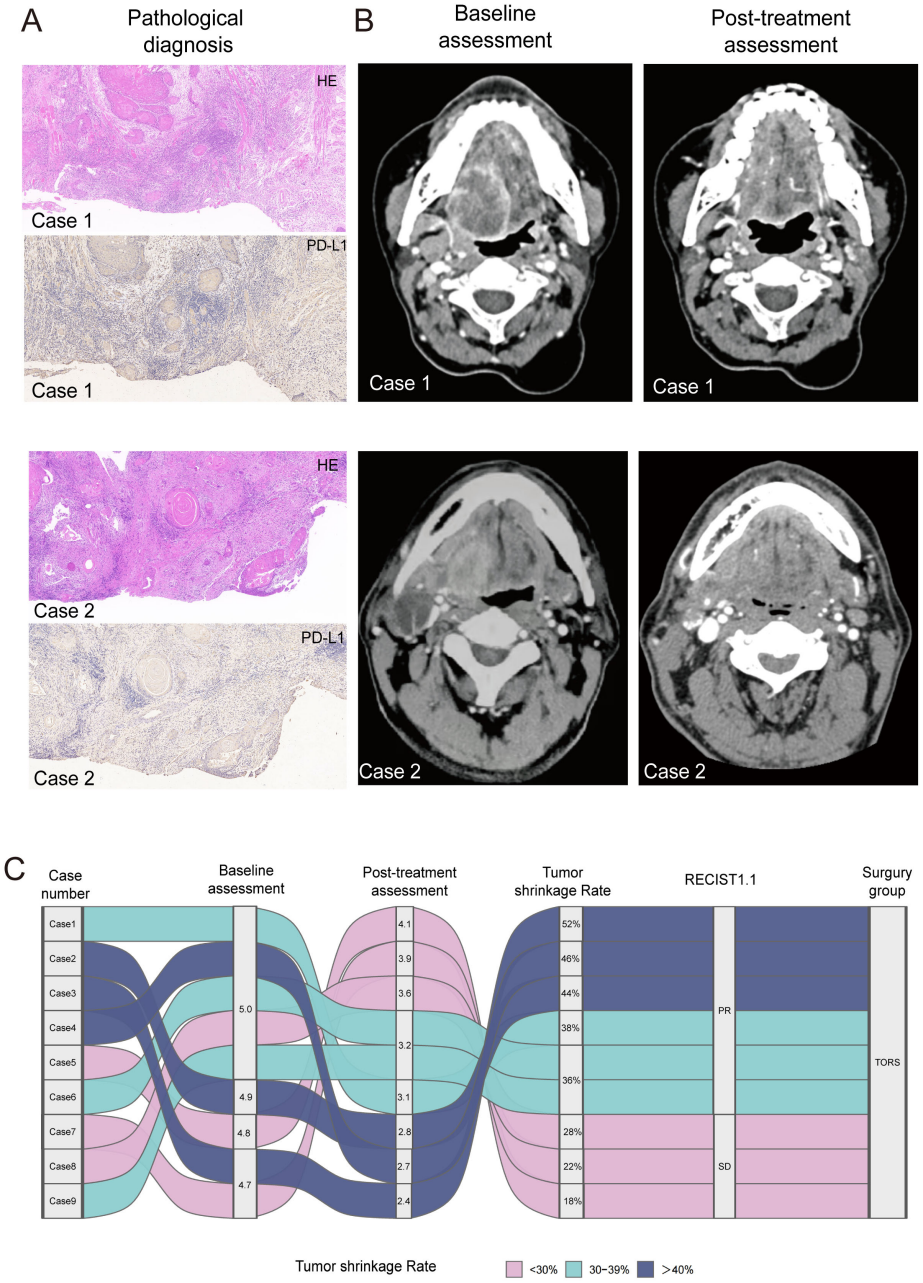
Pathology confirmation, radiologic response, and treatment flow after neoadjuvant immunotherapy. **(A)** Representative pre-treatment biopsies (Case 1 and Case 2). Hematoxylin–eosin (H&E) staining confirms base-of-tongue squamous cell carcinoma. PD-L1 immunohistochemistry (IHC) shows predominant immune-cell positivity with focal membranous staining on tumor cells, indicating an activated tumor immune microenvironment. **(B)** Axial contrast-enhanced CT at baseline and 1–2 weeks after completion of three cycles of pembrolizumab-based neoadjuvant therapy. Marked reduction of the primary lesion is seen in Case 1 (longest diameter 5.0 → 3.1; −38%) and Case 2 (4.9 → 2.8; −44%), improving exposure and margin definition for transoral resection. **(C)** Patient-level Sankey diagram summarizing baseline longest diameter, post-treatment longest diameter, percent tumor shrinkage, RECIST v1.1 assessment, and surgical approach. Ribbon width denotes the number of cases; color encodes shrinkage strata:<30% (pink), 30%–39% (teal), and ≥40% (navy). All patients proceeded to transoral robotic surgery (TORS) after radiologic downstaging.

The final analytic cohort therefore comprised nine patients who completed pembrolizumab-based neoadjuvant immunochemotherapy followed by conversion TORS with concurrent neck dissection. They consisted of five male and four female patients, with a median age of 60 years (range, 55–68) and with advanced BOT-SCC (≥T4N2bM0). HPV was positive in five cases (55.6%), and the median PD-L1 CPS was 9 (IQR, 6–12). Ever-smoking was documented in 66.7% of nine cases and 55.5% of patients have ever alcohol use. All patients received the full course of three neoadjuvant immunotherapy cycles. Subsequently, after documented downstaging on imaging, all patients underwent TORS and concurrent neck dissection. The median postoperative hospital stay was 7 days (IQR, 6–9; range, 5–10) (**Figure 2A**, **Table 2**).

**Table 2:** Baseline characteristics.

Patient	Sex	Age	T	N	M	p16 HPV	PD-L1 CPS	Smoking ever	Alcohol ever	Length of stay days
Case 1	M	55	T4	N1*	M0	POS	14	Yes	No	7
Case 2	F	56	T4	N2c^†^	M0	NEG	18	No	Yes	6
Case 3	F	57	T4	N1*	M0	POS	20	No	No	6
Case 4	M	58	T4	N3*	M0	POS	15	Yes	Yes	5
Case 5	M	60	T4	N2b^†^	M0	NEG	1	Yes	Yes	10
Case 6	F	61	T4	N1*	M0	POS	10	Yes	No	7
Case 7	M	62	T4	N2b^†^	M0	NEG	1	Yes	Yes	9
Case 8	F	65	T4	N3^†^	M0	NEG	4	No	No	8
Case 9	M	68	T4	N2*	M0	POS	5	Yes	Yes	6

*N category based on AJCC 8th edition staging for HPV-mediated (p16-positive) oropharyngeal cancer; ^†^N category based on AJCC 8th edition staging for HPV-negative oropharyngeal cancer.

*N categories for p16-positive tumors (POS) were assigned according to the AJCC 8th edition HPV-mediated (p16-positive) oropharyngeal carcinoma chapter (N1 = one or more ipsilateral lymph nodes, none >6 cm; N2 = contralateral or bilateral lymph nodes, none >6 cm; N3 = lymph node >6 cm). N categories for p16-negative tumors (NEG, ^†^) follow the AJCC 8th edition conventional oropharyngeal carcinoma chapter (N2b = multiple ipsilateral lymph nodes ≤6 cm; N2c = bilateral or contralateral lymph nodes ≤6 cm; N3 = lymph node >6 cm or with extranodal extension).

PD-L1 CPS = (PD-L1-positive tumor and immune cells/viable tumor cells) ×100.

Smoking/Alcohol (ever) includes current or former use.

### 3.2 Radiologic response

Contrast-enhanced CT was performed at baseline and repeated 1–2 weeks after the completion of three cycles of neoadjuvant immunotherapy (**Figure 2B**).

According to RECIST 1.1 criteria, six of the nine patients (66.7%) achieved a PR, and three (33.3%) had SD. The ORR was 66.7%; no CR or progressive disease (PD) was observed. The mean tumor shrinkage was 35.6% ± 11.2%, with a median reduction of 36% (IQR, 28–44; range, 18%–52%). HPV-positive tumors exhibited a greater degree of shrinkage compared to HPV-negative cases (mean 41.6% *vs*. 28.0%), suggesting a potential association between HPV status and treatment response (**Figure 2C**, **Table 3**).

**Table 3:** Imaging evaluation results after neoadjuvant therapy.

Patients	SLD*_pre_*	SLD*_post_*	Shrinkage rate (%)	Efficacy
Case 1	5	3.1	38	PR
Case 2	4.9	2.8	44	PR
Case 3	4.7	2.4	52	PR
Case 4	5	2.7	46	PR
Case 5	4.8	4.1	18	SD
Case 6	5	3.2	36	PR
Case 7	4.7	3.9	22	SD
Case 8	5	3.6	28	SD
Case 9	5	3.2	36	PR

Representative radiologic outcomes following neoadjuvant therapy included a reduction in the primary lesion from 5.0 to 3.1 cm in Case 1 (−38%; PR per RECIST) and from 5.0 to 3.6 cm in Case 2 (−44%; PR per RECIST).

### 3.3 Toxicities during neoadjuvant therapy

In the safety set (*n* = 9), no immune-mediated adverse events were observed during neoadjuvant pembrolizumab, including pneumonitis, immune-related colitis/diarrhea, hepatitis, thyroid dysfunction (hypo/hyperthyroidism/thyroiditis), hypophysitis/adrenal insufficiency/type 1 diabetes, nephritis, or immune-mediated rash/dermatitis [all 0/9; 0.0% (95% CI: 0–33.6)]. No infusion reactions or hypersensitivity reactions occurred [0/9; 0.0% (0–33.6)]. Hematuria was not recorded [0/9; 0.0% (0–33.6)].

The most frequent non-immune AEs were rash and pruritus [each 4/9; 44.4% (95% CI: 13.7–78.8)], followed by fatigue [3/9; 33.3% (7.5–70.1)], nausea [2/9; 22.2% (2.8–60.0)], and non-immune diarrhea [1/9; 11.1% (0.3–48.2)]; fever was not observed [0/9; 0.0% (0–33.6)]. No AE led to postponement or cancellation of TORS, and none impacted intra-operative management (**Table 4**).

**Table 4:** Toxicities during neoadjuvant therapy.

Category	Category	*n*/9	% (95% CI)	Impact on surgery
Immune-related	Pneumonitis	0/9	0.0% (0–33.6)	No
	Colitis/diarrhea	0/9	0.0% (0–33.6)	No
	Hepatitis	0/9	0.0% (0–33.6)	No
	Hypothyroid-related	0/9	0.0% (0–33.6)	No
	Nephritis	0/9	0.0% (0–33.6)	No
	Rash/dermatitis	0/9	0.0% (0–33.6)	No
	Infusion-related	0/9	0.0% (0–33.6)	No
	Hypersensitivity	0/9	0.0% (0–33.6)	No
	Hematuria	0/9	0.0% (0–33.6)	No
Non-immune	Fatigue	3/9	33.3% (7.5–70.1)	No
	Diarrhea	1/9	11.1% (0.3–48.2)	No
	Nausea	2/9	22.2% (2.8–60.0)	No
	Rash	4/9	44.4% (13.7–78.8)	No
	Pruritus	4/9	44.4% (13.7–78.8)	No
	Fever	0/9	0.0% (0–33.6)	No

### 3.4 Neck dissection

Neck dissection was performed concurrently with TORS in all patients. The median total lymph-node yield was 26 (IQR, 24–29), and 5/9 (55.6%, 95% CI: 21.2–86.3) patients were node-positive. Among node-positive cases, the median number of positive nodes was 1 (range, 1–2). Dissection side was left in 6/9 (66.7%, 95% CI: 29.9–92.5) and right in 3/9 (33.3%, 95% CI: 7.5–70.1). Final nodal staging (AJCC 8th) was pN0 4/9 (44.4%, 95% CI: 13.7–78.8), pN1 4/9 (44.4%, 95% CI:13.7–78.8), and pN2 1/9 (11.1%, 95% CI: 0.3–48.2) (**Table 5**).

**Table 5:** Cervical lymph nodes.

Patients	Side	Levels	Total LN	Positive LN	pN (AJCC8)
Case 1	Left	Ia Ib II–V	24	1	pN1
Case 2	Left	Ia Ib II–V	28	1	pN1
Case 3	Left	Ia Ib II–V	30	0	pN0
Case 4	Left	Ia Ib II–V	32	0	pN0
Case 5	Right	Ia Ib II–V	26	2	pN2
Case 6	Right	Ia Ib II–V	22	0	pN0
Case 7	Left	Ia Ib II–V	23	1	pN1
Case 8	Left	Ia Ib II–V	25	1	pN1
Case 9	Right	Ia Ib II–V	27	0	pN0

### 3.5 Surgical outcomes

#### 3.5.1 Intraoperative metrics

Following radiologic downstaging with neoadjuvant immunotherapy, all patients successfully underwent TORS with concurrent neck dissection. An R0 resection was achieved in all cases. The mean operative time was 193.3 ± 46.9 min (median, 180; IQR, 165–240; range, 125–255), and the mean estimated blood loss was 78.3 ± 25.4 mL (median, 70; IQR, 60–100; range, 50–120). Tumor shrinkage demonstrated a strong inverse correlation with operative time and a moderate-to-strong inverse correlation with blood loss. A positive correlation was also observed between operative time and blood loss. Together, these findings suggest that greater tumor regression after neoadjuvant therapy is associated with shorter operative duration and reduced intraoperative bleeding (**Table 6**, **Supplementary Table 2**).

**Table 6:** Quantified table of Operative time and intraoperative bleeding.

Patients	Operative time (min)	Intraoperative bleeding (mL)	R0 resection	Pearson *r*	*p*	95% CI
Case 1	180	60	Yes			0.469–0.971
Case 2	165	55	Yes			
Case 3	125	50	Yes			
Case 4	140	75	Yes			
Case 5	245	100	Yes	0.864	0.003	
Case 6	180	65	Yes			
Case 7	255	120	Yes			
Case 8	240	110	Yes			
Case 9	210	70	Yes			

#### 3.5.2 Association analysis

Pearson correlation analysis revealed a strong inverse association between tumor shrinkage and operative time (*r* = −0.962; 95% CI: −0.992 to −0.825; *p* < 0.001), as well as a significant inverse correlation between tumor shrinkage and intraoperative blood loss (*r* = −0.851; 95% CI: −0.968 to −0.430; *p* = 0.004). A positive correlation was also observed between operative time and blood loss (*r* = 0.864; 95% CI: 0.469 to 0.971; *p* = 0.003). In this cohort, PD-L1 CPS was positively correlated with tumor shrinkage (*r* = 0.941; 95% CI: 0.739 to 0.988; *p* < 0.001) and negatively correlated with both operative time (*r* = −0.952; 95% CI −0.990 to −0.783; *p* < 0.001) and blood loss (*r* = −0.861; 95% CI: −0.970 to −0.458; *p* = 0.003) (**Supplementary Table 2**).

Stratified analyses further demonstrated that perioperative blood loss was significantly lower in the PR group compared to the SD group (60.0 ± 9.6 mL *vs*. 101.3 ± 23.1 mL; 95% CI: −4.38 to −0.58; *p* = 0.0176). Operative time was shorter in the PR group, although the difference did not reach statistical significance (176.7 ± 33.1 min *vs*. 225.0 ± 49.3 min; 95% CI: −2.62 to 0.39; *p* = 0.178). HPV-positive cases also showed a trend toward shorter operative times (167.0 ± 34.2 min *vs*. 226.3 ± 40.0 min; 95% CI: −2.95 to 0.08; *p* = 0.062) and reduced blood loss (64.0 ± 9.3 mL *vs*. 96.3 ± 29.7 mL; 95% CI: −2.89 to 0.11; *p* = 0.106) compared to HPV-negative cases (**Table 7**).

**Table 7:** Group comparison.

Group		*n*	Operative time	*p*-value	95% CI	Intraoperative bleeding (mL)	*p*-value	95% CI
Response	PR	6	176.7 ± 33.1	0.178	−2.62 to 0.39	60.0 ± 9.6	0.0176	−4.38 to −0.58
	SD	3	225.0 ± 49.3			101.3 ± 23.1		
HPV	Positive	5	167.0 ± 34.2	0.062	−2.95 to 0.08	64.0 ± 9.3	0.106	−2.89 to 0.11
	Negative	4	226.3 ± 40.0			96.3 ± 29.7		

### 3.6 Pathological response

All resected specimens were histologically confirmed as BOT-SCC. Following neoadjuvant therapy, all nine patients underwent TORS with concurrent neck dissection and achieved R0 resection.

PD-L1 immunohistochemistry revealed predominant immune cell positivity accompanied by focal membranous staining on tumor cells, indicative of an activated tumor immune microenvironment. All nine cases satisfied the criteria for MPR.

**Figure 3:**
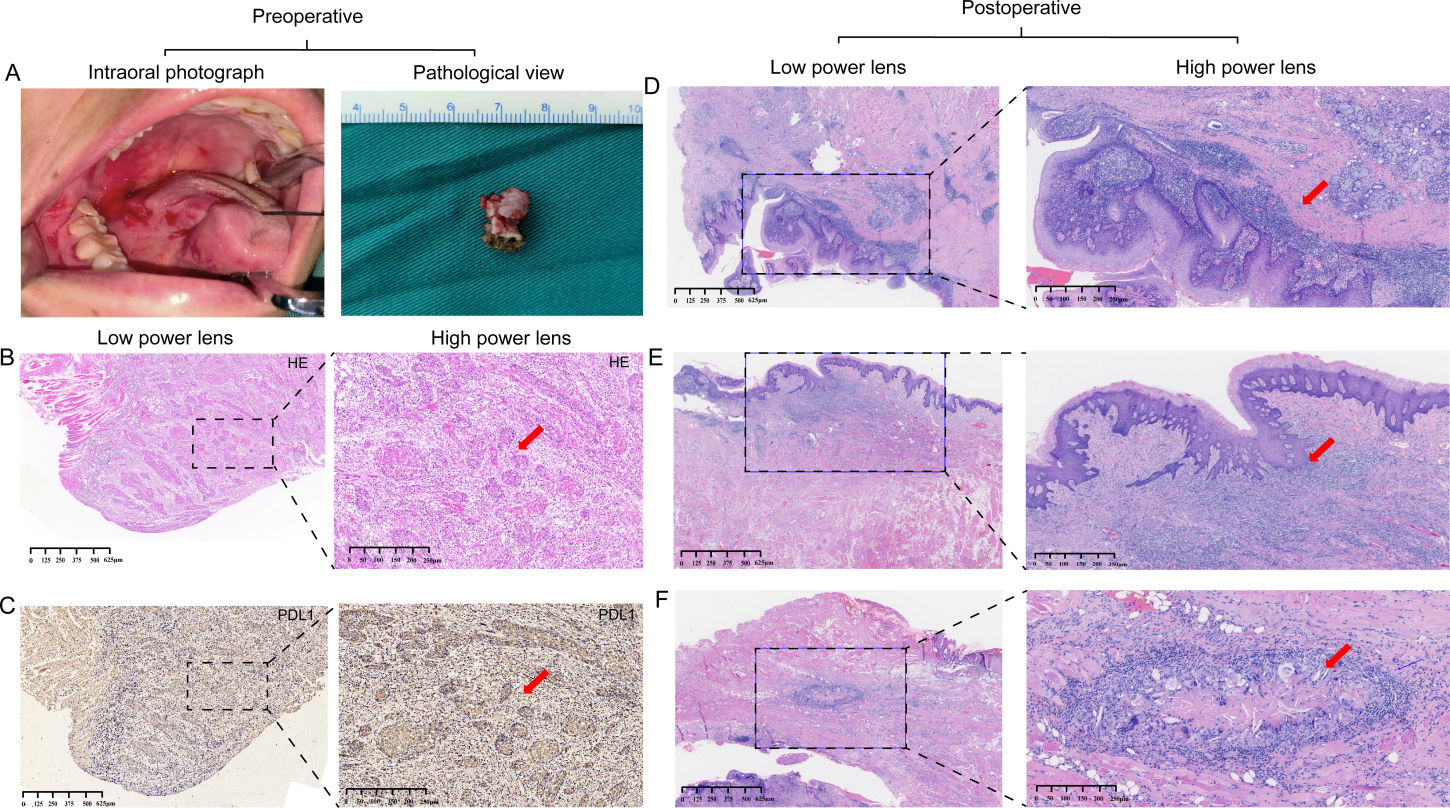
Representative clinical, histologic, and immunophenotypic findings before and after neoadjuvant immunotherapy in BOT-SCC. **(A)** Left: intraoral photograph showing an exophytic lesion at the right base of tongue. Right: gross view of the pretreatment biopsy specimen. **(B)** Pretreatment histology (H&E). Left: low-power view demonstrating infiltrative nests of keratinizing squamous cell carcinoma. Right: high-power view highlighting well-differentiated SCC with keratinization and intercellular bridges (red arrow). **(C)** Diffuse PD-L1 positivity in immune cells with focal membranous staining of tumor cells (red arrow), indicating an activated tumor immune microenvironment. **(D)** Low- and high-power views showing surface re-epithelialization and reactive epithelial hyperplasia (red arrow). **(E)** Low- and high-power views showing dense subepithelial chronic inflammatory infiltrates with lymphoid aggregates/tertiary lymphoid structures (red arrow). **(F)** Low- and high-power views showing multinucleated giant-cell reaction with stromal remodeling and capillary neovascularization (red arrow).

Histological evaluation of the resection specimens showed that the original tumor bed was largely replaced by fibrotic and hyalinized tissue with granulation tissue formation, surface re-epithelialization, and reactive hyperplasia. Dense lymphoplasmacytic infiltrates were present, frequently organized into tertiary lymphoid structures (TLS), accompanied by a multinucleated giant-cell reaction and capillary neovascularization. No cohesive, structurally invasive nests of viable carcinoma were identified in the examined sections, and mitotic figures were rare. Collectively, these histopathological features are consistent with an immune-related pathologic response (irPR) (**Figure 3**).

### 3.7 Postoperative follow-up

#### 3.7.1 Functional outcome assessment

At the 3-month postoperative follow-up, outpatient functional assessments, including the MDADI, FOIS, and GRBAS scale, were completed. The mean global MDADI score was 75.6 ± 10.2 (median, 74; IQR, 68–84; range, 60–88). Five of the nine patients (55.6%) achieved an FOIS score ≥6, corresponding to a regular or near-regular diet, while the remaining four patients scored between 3 and 5, indicating partial dependence on tube feeding and/or modified oral intake. The mean GRBAS score was 1.24 ± 0.55 (median, 1.1; IQR, 1.0–1.6; range, 0.5–2.0), reflecting predominantly mild-to-moderate dysphonia, with daily communication largely preserved. In comparison with historical cohorts treated with open surgery for advanced BOT-SCC, these outcomes suggest superior early preservation of swallowing and vocal function (**Tables 8**, **9**).

**Table 8:** Postoperative functional recovery indicators (3 months after surgery).

Patients	MDADI	FOIS	GRBAS
Case 1	80	6	0.5
Case 2	84	6	1
Case 3	88	7	1.1
Case 4	88	6	0.6
Case 5	60	5	1
Case 6	64	4	1.4
Case 7	68	3	1.6
Case 8	74	4	2
Case 9	74	6	2

**Table 9:** Postoperative functional recovery indicators (3 months after surgery).

Indicators	Mean ± SD	Median [IQR]	Range
MDADI	75.6 ± 10.2	74 [68–84]	60–88
FOIS	5.1 ± 1.4	6 [4–6]	3–7
GRBAS	1.24 ± 0.55	1.1 [0.8–1.6]	0.5–2.0
FOIS ≥ 6 *n* (%)	-	5 (55.6%)	-

#### 3.7.2 Survival

At a median follow-up of 12 months from surgery (range, 8–15 months), no local recurrences or distant metastases were observed. Accordingly, the 12-month DFS and OS rates were both 100% (**Table 10**).

**Table 10:** DFS and OS.

Patients	Follow-up time (months)	Recurrence	Status
Case 1	15	No	Alive
Case 2	9	No	Alive
Case 3	10	No	Alive
Case 4	11	No	Alive
Case 5	12	No	Alive
Case 6	12	No	Alive
Case 7	13	No	Alive
Case 8	14	No	Alive
Case 9	8	No	Alive

## 4 Discussion

In this retrospective study, patients with advanced BOT-SCC who were downstaged by neoadjuvant immunotherapy and conversion to TORS demonstrated feasibility and short-term benefit. All nine patients underwent TORS with concurrent neck dissection and achieved R0 resection without severe perioperative complications. Radiographic ORR was 66.7% with a median tumor shrinkage of 36%, and there were no recurrences and deaths during a median 12-month follow-up.

Compared with the existing literature, our findings should be interpreted alongside contemporary reports of neoadjuvant immunotherapy in resectable or locally advanced BOT-SCC and organ-preserving approaches. While direct cross-trial comparisons are inappropriate, our objective radiologic responses and pathologic regression signals appear within the range reported for similar regimens in head and neck cancer and align with the concept that selected tumors may be downstaged into safe transoral resection (18).

### 4.1 Efficacy of neoadjuvant immunotherapy

After three cycles of neoadjuvant immunotherapy, 66.7% of patients achieved PR, with mean shrinkage (35.6%) consistent with prior reports in resectable or locally advanced HNSCC that document meaningful pathologic response after perioperative PD-1 inhibitor (19). Clinically, reduced tumor bulk improved exposure and margin delineation within the constrained oropharyngeal corridor, enabling safe transoral resection. The 100% R0 resection rate supports the practical value of this approach.

At the same time, we also found the occurrence of adverse reactions in accordance with previous reports, such as rash, but no adverse reactions affect the surgical treatment. We will further refine the reported adverse effect data in a larger, multicenter study (20–24).

### 4.2 Surgical feasibility and oncologic adequacy

TORS procedures were stable and low morbidity (operative time, 193.3 ± 46.9 min; blood loss,78.3 ± 25.4 mL). Tumor shrinkage correlated strongly and inversely with operative time (*r* = −0.962; 95% CI: −0.992 to −0.825; *p* < 0.001) and moderately to strongly with reduced blood loss (*r* = −0.851; 95% CI: −0.968 to −0.430; *p* = 0.004), while operative time correlated positively with blood loss (*r* = 0.864; 95% CI: 0.469 to 0.971; *p* = 0.003). These associations indicate that greater regression after neoadjuvant immunotherapy facilitates exposure and dissection, translating into shorter procedures and less intraoperative bleeding (16, 17). From a conversion-surgery perspective, these quantitative links provide mechanistic support for improved operability and margin control following neoadjuvant immunotherapy (25).

Among the patients receiving neoadjuvant adjuvant therapy, we also found patients with poor immune response, but because of the small sample size, we did not discuss it in detail here. These are worthy of future investigation.

### 4.3 Early functional recovery and quality of life

At 3 months postoperatively, functional recovery was generally favorable: mean MDADI 75.6 ± 10.2 (median, 74; IQR, 68–84), FOIS ≥ 6 in 55.6%, and median GRBAS 1.24 ± 0.55 (median, 1.1; IQR, 1.0–1.6). Although this study lacked a control group, these results fall within reported TORS ranges and align with the premise that transoral, minimally invasive surgery supported organ and function preservation. In clinical practice, the “neoadjuvant immunotherapy with TORS” pathway achieved disease control while reducing the likelihood of long-term tube dependence and clinically significant dysphonia (13, 26, 27).

### 4.4 Biological and stratification signals

Exploratory analyses suggested lower blood loss and a trend toward shorter operative time in patients achieving PR compared with SD. HPV-positive status and higher PD-L1 CPS correlated with greater shrinkage and more favorable intraoperative metrics, indicating the potential predictive value of these biomarkers (12, 28). These findings are hypothesis-generating and require larger prospective studies to develop predictive models for “conversion success” (29).

### 4.5 Perioperative safety

No Clavien–Dindo grade ≥III complications or postoperative hemorrhage occurred (23). Although variable inflammatory-fibrotic changes were encountered after neoadjuvant immunotherapy, tissue planes generally remained operable and did not increase perioperative risk when standard perioperative management and appropriate drug-to-surgery intervals were observed (30).

### 4.6 Limitations and outlook

This single-center, retrospective study has several limitations, including small sample size and potential selection bias. We explicitly acknowledge that the median follow-up of 12 months is relatively short. Consequently, the reported oncologic outcomes should be interpreted as preliminary, and ongoing surveillance is in progress. This follow-up duration also limits the assessment of long-term disease control and functional durability. Methodological rigor in future studies could be enhanced through centralized imaging and pathology reviews, as well as the inclusion of objective functional endpoints, such as instrumental swallowing evaluation (31–33).

Accordingly, we present effect sizes with 95% CIs, treat *p*-values as descriptive, and refrain from efficacy claims. Larger prospective, multi-institutional studies with standardized imaging and pathology endpoints and predefined functional benchmarks are warranted to test durability and external validity.

Future investigations should employ prospective, multicenter designs with predefined endpoints (e.g., MPR, feeding-tube dependence, possibilities for radiotherapy de-intensification, and long-term quality of life). Furthermore, validating immunologic biomarkers (e.g., HPV status and PD-L1 CPS) to predict successful conversion and guide treatment de-intensification is warranted. The evaluation of individualized strategies regarding the extent of neck dissection and adjuvant radiotherapy dosing, while preserving oncologic safety, is also merited (33–35).

## 5 Conclusion

With careful patient selection and standardized perioperative protocols, neoadjuvant immunotherapy facilitated conversion TORS in a subset of patients with advanced BOT-SCC, resulting in R0 resections with encouraging early functional preservation and low perioperative morbidity. However, given the small sample size, single-center retrospective design, and the median follow-up of 12 months, these findings must be regarded as preliminary. Nonetheless, the conversion TORS approach following neoadjuvant immunotherapy demonstrates feasibility and an acceptable safety profile, justifying its further validation in larger, prospective, multicenter studies with predefined endpoints and longer follow-up.

## Data Availability

The original contributions presented in the study are included in the article/**Supplementary Material**. Further inquiries can be directed to the corresponding authors.
