# Supplementary material for: Conversion TORS after neoadjuvant immunotherapy for advanced BOT-SCC: a retrospective study
Source: Front Oncol. 2026 Jan 6;15:1709974. doi: 10.3389/fonc.2025.1709974 (PMC12815781; doi:10.3389/fonc.2025.1709974)
Supplement: Supplementary file 1 [file Table1.docx]

**Supplement Table 1. Patients detail information**

| **Patient** | **Evaluated at MDT** | **Eligible for neoadjuvant conversion TORS** | **Reason for exclusion or declining** | **Received neoadjuvant immunochemotherapy** | **Final primary treatment** | **Included in analytic cohort** |
| --- | --- | --- | --- | --- | --- | --- |
| Case1 | Yes | Yes |  | Yes | Neoadjuvant immunochemotherapy + conversion TORS + neck dissection | Yes |
| Case2 | Yes | Yes |  | Yes | Neoadjuvant immunochemotherapy + conversion TORS + neck dissection | Yes |
| Case3 | Yes | Yes |  | Yes | Neoadjuvant immunochemotherapy + conversion TORS + neck dissection | Yes |
| Case4 | Yes | Yes |  | Yes | Neoadjuvant immunochemotherapy + conversion TORS + neck dissection | Yes |
| Case5 | Yes | Yes |  | Yes | Neoadjuvant immunochemotherapy + conversion TORS + neck dissection | Yes |
| Case6 | Yes | Yes |  | Yes | Neoadjuvant immunochemotherapy + conversion TORS + neck dissection | Yes |
| Case7 | Yes | Yes |  | Yes | Neoadjuvant immunochemotherapy + conversion TORS + neck dissection | Yes |
| Case8 | Yes | Yes |  | Yes | Neoadjuvant immunochemotherapy + conversion TORS + neck dissection | Yes |
| Case9 | Yes | Yes |  | Yes | Neoadjuvant immunochemotherapy + conversion TORS + neck dissection | Yes |
| Case10 | Yes | No | Prior definitive treatment for index tumor | No | Not applicable (excluded from neoadjuvant conversion-TORS pathway) | No |
| Case11 | Yes | No | Documented distant metastases | No | Not applicable (excluded from neoadjuvant conversion-TORS pathway) | No |
| Case12 | Yes | No | Significant comorbidities precluding neoadjuvant therapy or surgery | No | Not applicable (excluded from neoadjuvant conversion-TORS pathway) | No |
| Case13 | Yes | No | High risk of airway obstruction | No | Not applicable (excluded from neoadjuvant conversion-TORS pathway) | No |
| Case14 | Yes | No | Prior definitive treatment for index tumor | No | Not applicable (excluded from neoadjuvant conversion-TORS pathway) | No |
| Case15 | Yes | Yes | Declined neoadjuvant therapy and preferred definitive chemoradiation | No | Definitive concurrent chemoradiation | No |
| Case16 | Yes | Yes | Declined TORS and preferred definitive chemoradiation | No | Definitive concurrent chemoradiation | No |
| Case17 | Yes | Yes | Declined TORS and preferred primary open surgery | No | Primary open surgery + neck dissection | No |
| Case18 | Yes | Yes | Declined TORS and preferred primary open surgery | No | Primary open surgery + neck dissection | No |

**Supplement Table 2. Association analysis**

**
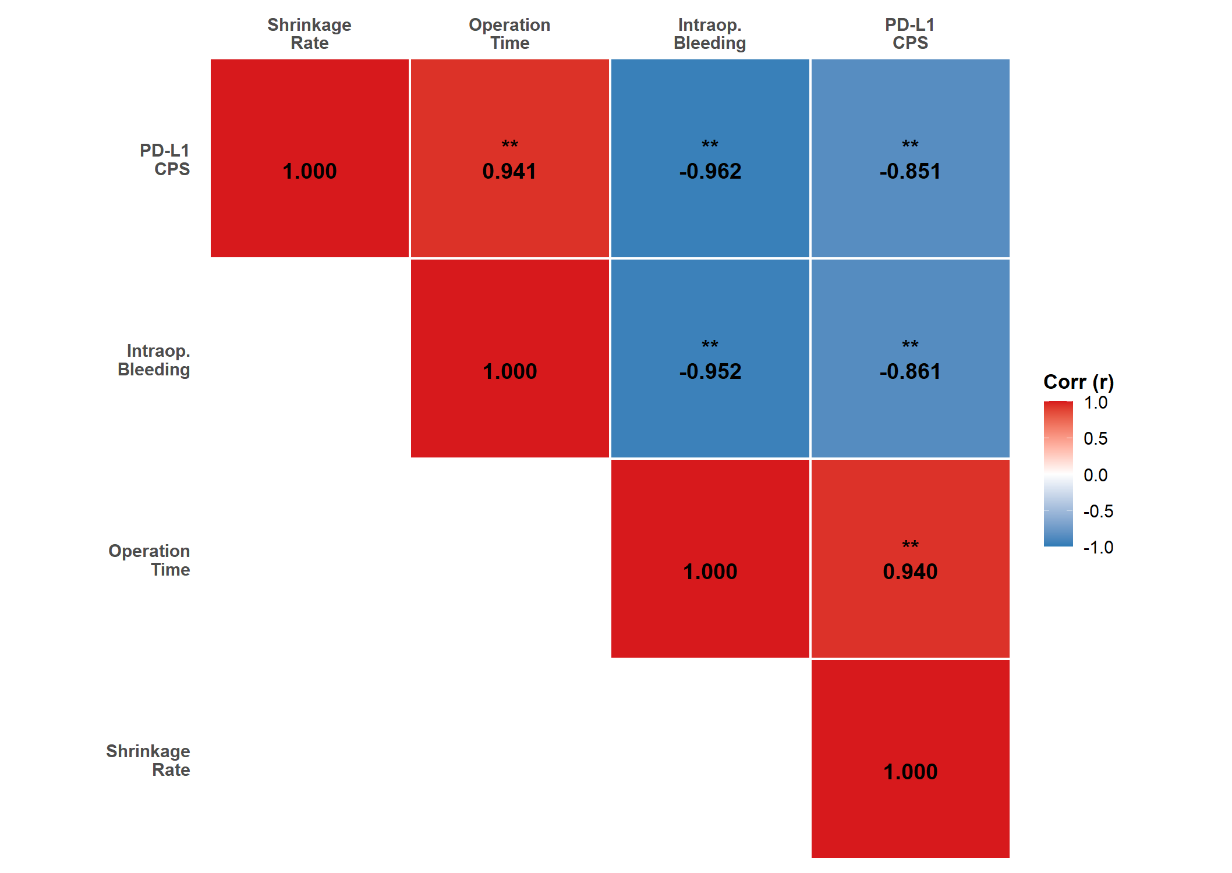
**
